# Do Price Subsidies on Artemisinin Combination Therapy for Malaria Increase Household Use?: Evidence from a Repeated Cross-Sectional Study in Remote Regions of Tanzania

**DOI:** 10.1371/journal.pone.0070713

**Published:** 2013-07-29

**Authors:** Jessica L. Cohen, Prashant Yadav, Corrina Moucheraud, Sarah Alphs, Peter S. Larson, Jean Arkedis, Julius Massaga, Oliver Sabot

**Affiliations:** 1 Global Health and Population Department, Harvard School of Public Health, Harvard University, Boston, Massachusetts, United States of America; 2 Global Economy and Development, Brookings Institution, Washington, DC, United States of America; 3 William Davidson Institute, Ross School of Business and School of Public Health, University of Michigan, Ann Arbor, Michigan, United States of America; 4 Results for Development Institute, Washington, DC, United States of America; 5 National Institute for Medical Research, Dar es Salaam, Tanzania; 6 Slingshot, London, United Kingdom; Tulane University School of Public Health and Tropical Medicine, United States of America

## Abstract

**Background:**

The Affordable Medicines Facility-malaria (AMFm) is a pilot program that uses price subsidies to increase access to Artemisinin Combination Therapies (ACTs), currently the most effective malaria treatment. Recent evidence suggests that availability and affordability of ACTs in retail sector drug shops (where many people treat malaria) has increased under the AMFm, but it is unclear whether household level ACT use has increased.

**Methods and Findings:**

Household surveys were conducted in two remote regions of Tanzania (Mtwara and Rukwa) in three waves: March 2011, December 2011 and March 2012, corresponding to 3, 13 and 16 months into the AMFm implementation respectively. Information about suspected malaria episodes including treatment location and medications taken was collected. Respondents were also asked about antimalarial preferences and perceptions about the availability of these medications. Significant increases in ACT use, preference and perceived availability were found between Rounds 1 and 3 though not for all measures between Rounds 1 and 2. ACT use among suspected malaria episodes was 51.1% in March 2011 and increased by 10.9 percentage points by Round 3 (p = .017). The greatest increase was among retail sector patients, where ACT use increased from 31% in Round 1 to 49% in Round 2 (p = .037) and to 61% (p<.0001) by Round 3. The fraction of suspected malaria episodes treated in the retail sector increased from 30.2% in Round 1 to 46.7% in Round 3 (p = .0009), mostly due to a decrease in patients who sought no treatment at all. No significant changes in public sector treatment seeking were found.

**Conclusions:**

The AMFm has led to significant increases in ACT use for suspected malaria, especially in the retail sector. No evidence is found supporting the concerns that the AMFm would crowd out public sector treatment or neglect patients in remote areas and from low SES groups.

## Introduction

Artemisinin-based combination therapies (ACTs) are currently the most effective medication for the treatment of *Plasmodium falciparum* malaria and are the first-line treatment recommended by the WHO [1]. National programs scaling up access to ACTs have been linked to substantial reductions in malaria morbidity and mortality [2], [3]. Despite the fact that this recommendation has been adopted in the national malaria treatment guidelines in most countries with malaria, access to ACTs has been limited due to a number of factors including, but not limited to, high prices in the private sector [4]-[6], frequent stock outs in the public sector [7]-[10], and challenges in reaching remote populations [11], [12].

In 2010, the Global Fund to Fight AIDS, TB and Malaria launched the Affordable Medicines Facility for malaria (AMFm) as a pilot program in seven countries. The AMFm offers public and private sector first-line buyers access to heavily subsidized ACTs, with a primary objective of making low-priced, quality-assured [13] ACTs widely available through private sector establishments [14], [15]. Participating countries were also required to implement supporting interventions (such as awareness campaigns) to increase the availability and use of the subsidized ACTs. As the private sector is a common source of malaria treatment [5], [16], by encouraging wide-scale availability and awareness of low-priced, over-the-counter ACTs in private outlets, the AMFm aims to increase the use of ACTs at the expense of older, less-effective treatments and artemisinin monotherapies [14], [15], [17]. The AMFm is innovative in its leveraging of the private sector, but is an unproven approach to scaling up effective malaria treatment. Encouraging evidence is emerging that price subsidies do lead to increased availability and lower prices of private sector ACTs [18], [19], but it is unclear to what extent this translates into increases in household level use of ACTs. ACT use could remain unchanged if malaria patients are unaware that ACTs are the most effective antimalarials, simply prefer the older antimalarials or if, even in the presence of subsidies, ACTs remain financially out-of-reach [20], [21]. These barriers to ACT use could be particularly important in rural, remote regions [11], [12]. Finally, if malaria patients seeking ACTs simply switch from public sector to private sector treatment in the presence of ACT subsidies, the AMFm could have a negligible impact on overall ACT use.

This study used repeated cross-sectional data collected during the first 1.5 years of the AMFm in Tanzania to assess trends in ACT use. Data from periodic household surveys conducted in two remote regions of Tanzania are used to assess the impact of the AMFm on malaria treatment seeking, antimalarial purchases and awareness of and preferences for ACTs. This study is one of the first to explore whether a private sector subsidy for ACTs, which has led to a demonstrated increase in availability of low-priced ACTs, also results in increased use of ACTs for malaria treatment in remote regions of Africa.

## Methods

### Study Setting and Overview

**Figure 1:**
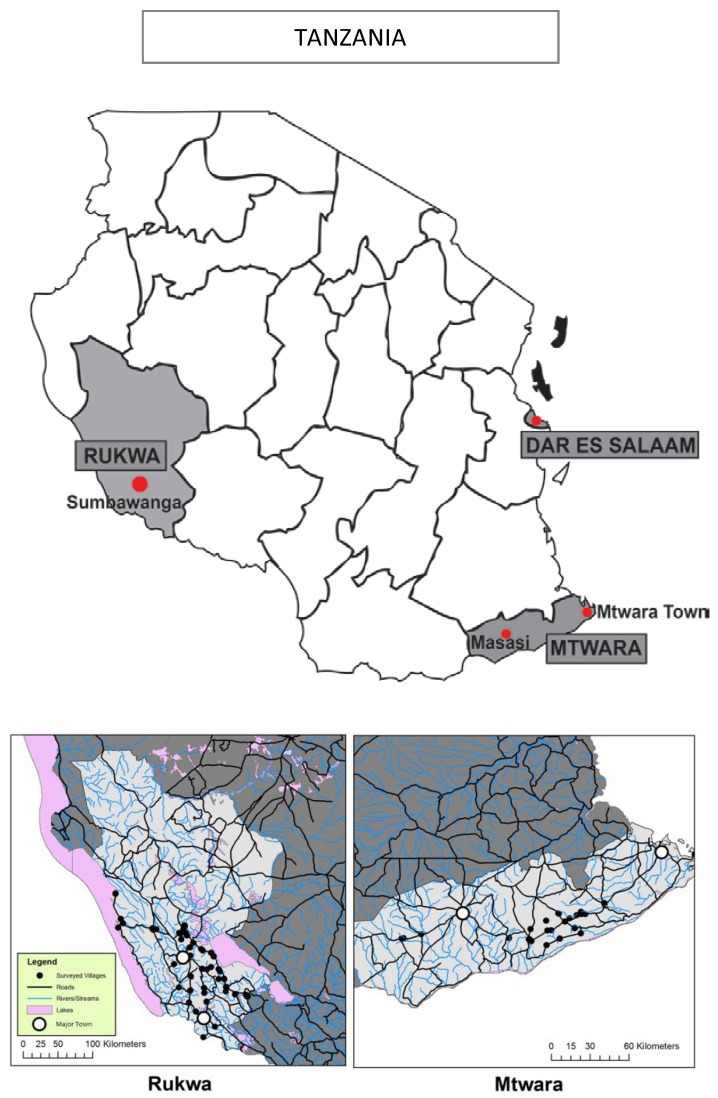
Map of the Two Study Regions Selected and Major Towns in Each Region.

The study took place in two regions of Tanzania - Mtwara and Rukwa (Figure 1) - chosen based on their remoteness, the absence of non-AMFm malaria interventions, and the presence of Accredited Drug Dispensing Outlets (ADDOs). ADDOs are licensed, accredited retail drug outlets approved by the Tanzania Food and Drug Authority and now present in most regions of Tanzania. Malaria prevalence estimates in Rukwa and Mtwara are roughly 7% and 18%, respectively [22]. Both regions are predominantly rural with the majority of the population engaged in agriculture. Rukwa is one of the largest and lowest population density regions in Tanzania and is more remote than Mtwara, with a distance between Sumbawanga (Rukwa’s regional headquarters and main urban center) and Dar es Salaam (1150 km) more than twice the distance between Mtwara’s main urban center and Dar es Salaam (556 km) [23].

**Figure 2:**
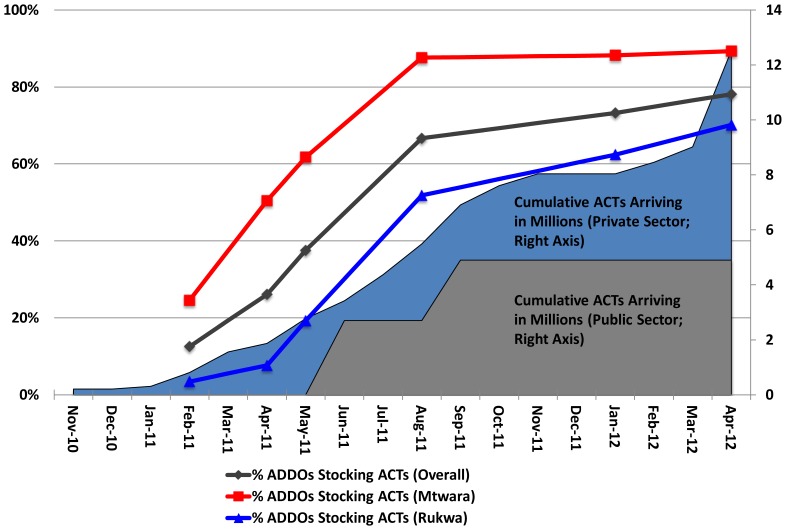
ACT Deliveries, Retail Outlet ACT Stocking and Survey Timing.

This research was part of a larger study with a longitudinal component consisting of ADDO surveys (“retail audits”) and customer exit interviews at ADDOs in the two regions, and with a repeated cross-sectional component consisting of household surveys in the catchment area of these ADDOs. The data collection period extended from February 2011 (three months after ACTs arrived in Tanzania) through May 2012 and included three rounds of household surveys, occurring in February/March 2011, November/December 2011 and March 2012 (Figure 2). Methods for the retail audits and exit interviews are described elsewhere [24]. Survey rounds were originally planned at equally-spaced intervals but Round 3 was accelerated in order to deliver results in time for the independent evaluation of the AMFm.

The first AMFm shipment arrived in Tanzania in November 2010 (Figure 2) and ACT orders grew rapidly between 2011 and the first half of 2012, from roughly 300,000 subsidized ACT treatments delivered in January 2011 to 29.1 million subsidized ACT treatments approved and 21.1 million treatments delivered by June 2012. On average, over one million treatments were approved each month over this period; however, no orders in June, July, or September 2011 were approved. Roughly 34% of the cumulative orders were for the Tanzanian public sector with the remaining 66% for the private, for-profit sector. Figure 2 also reproduces results on ACT stocking in ADDOs as reported in the companion paper by Yadav et al. (2012) [24]. The fraction of ADDOs stocking ACTs increased from 25% to 90% in Mtwara and 5% to 70% in Rukwa between January 2011 and May 2012. Supporting interventions in the form of television and radio advertisements and print media were scaled-up in early September 2011. By December 2011, 12,700 radio and television spots had been aired, 48 advertisements placed in newspapers, and several billboards created. Community outreach initiatives in 12 regions including the study regions of Mtwara and Rukwa also began in September 2011, emphasizing the availability and affordability of the subsidized ACTs to decision makers, caregivers of children under five, as well as the general community. In addition to these efforts, other NGOs and community-based organizations carried out broader campaigns throughout the country around case management and malaria treatment which could have increased the reach of the campaign.

### Sample Selection

Sample selection for household surveys was conducted as follows. First, 64 ADDOs were randomly selected among all ADDOs categorized as “remote” (based on distance to the nearest major trading center and major road) in Rukwa and Mtwara. Methods for categorizing remoteness are described in Yadav et al. (2012) [24]. Shop owners were asked to identify up to five nearby “Kitongojis” (sub-villages) from which their shop drew customers. From this list, one Kitongoji was randomly selected to conduct a complete household listing with village leaders. From these lists, 12 households per Kitongoji were randomly selected to be interviewed in each survey round. Households were sampled without replacement, so that once a household was selected for surveying in a given round it could not be surveyed again in future rounds. Of the 64 Kitongojis from which households were drawn, 42 were in Rukwa and 22 were in Mtwara. The reason for the imbalance across regions is that an additional region that borders Mtwara (Lindi region) was included in Round 1 but was dropped from Rounds 2 and 3 for budgetary reasons. Additionally, one Kitongoji in Rukwa was dropped for safety reasons. A total sample of 762 households was included in Round 1 and 756 households in Rounds 2 and 3, with a total of roughly 3900 individuals in each round.

### Data Collection

Informed consent was requested from, and household surveys were conducted with, the primary female caregiver in the household (the female responsible for most household healthcare) whenever possible. If the primary female caregiver was unavailable, an alternative respondent, over the age of 18 and knowledgeable about health-related decision making, was interviewed. The respondent was asked whether any member of the household had suspected malaria in the past four weeks and, if “yes”, the respondent was asked a series of questions about the steps taken to resolve each reported episode, including treatment location and medicines purchased. The survey recorded episodes of “suspected malaria”–that is, illnesses that the respondent or the patient believed were malaria and treated as such, regardless of whether the illness was diagnosed with a blood test or by a trained medical professional. For this reason, these episodes are referred to as “suspected malaria” throughout the text. Information on treatment location was recorded for the first action taken upon suspicion of malaria. The survey also included questions on knowledge about and preferences for antimalarials.

All data collection activities were conducted using electronic data collection tools and developed using Surveybe software Release 2.1 (Economic Development Initiatives Limited, High Wycombe United Kingdom).

### Analysis

Data were cleaned and analyzed using Stata Version 11.0 SE (Stata Corporation, College Station, TX). Variable means are presented as well as p-values on t-tests for differences in variable means across survey rounds. Results are unweighted and p-values are adjusted for clustering within Kitongojis. Given the number of reported suspected malaria episodes per round (see Table 1) and a baseline proportion of suspected malaria episodes treated with ACTs of roughly 50% (see Table 2), the minimum detectable effect size between Rounds 1 and 2 and Rounds 1 and 3 is 13 and 10 percentage points respectively, assuming 90% power and a 5% significance level. The study thus has more power to detect increases in ACT use between Rounds 1 and 3 than between Rounds 1 and 2. A socioeconomic status index was constructed based on available data on material assets. Information was recorded on possession of household items such as radios, television, livestock, house construction and types of toilet/water facilities (means of variables included in the SES index are presented in Table S1). All of these variables were dichotomized for presence/absence and used to create a principal components analysis based measure of socio-economic status [25]. This measure was then broken into tertiles to represent categories of economic status including “ultra poor,” “very poor,” and “poor.” SES tertiles were used–rather than the more conventional quintile categorization–because of limited sample size.

**Table 1:** Suspected Malaria Episodes (Experienced in the Past 4 Weeks) by Age, Region and SES.

	March 2011 (Round 1)			December 2011 (Round 2)		March 2012 (Round 3)		Change Round 1 to Round 2		Change Round 1 to Round 3	
	Total n		% Reporting Malaria	Total n	% ReportingMalaria	Total n	% ReportingMalaria	%	(p-value)	%	(p-value)
All Households	762		25.07	756	29.63	756	41.93	4.56	(0.0499)	16.87	(0.0000)
All Individuals	3,950		5.97	3,845	8.32	3,935	12.30	2.35	(0.0019)	6.33	(0.0000)
	Among Individuals										
*By Region*											
Rukwa	2,829	5.55		2,768	7.37	2,736	8.44	1.82	(0.0130)	2.89	(0.0006)
Mtwara	1,121	7.05		1,077	10.77	1,199	21.10	3.72	(0.0527)	14.05	(0.0000)
*By Age*											
Under 5 years	613	10.44		591	11.51	599	15.36	1.07	(0.5344)	4.92	(0.0343)
5 years and over	3,335	5.16		3,254	7.74	3,336	11.75	2.59	(0.0015)	6.59	(0.0000)
*By SES*											
Bottom Tertile	1,318	5.61		1,284	6.23	1,316	12.39	0.62	(0.5179)	6.77	(0.0000)
Middle Tertile	1,314	5.94		1,278	9.23	1,310	12.90	3.30	(0.0084)	6.96	(0.0000)
Upper tertile	1,318	6.37		1,283	9.51	1,309	11.61	3.14	(0.0305)	5.24	(0.0020)

**Table 2:** ACT Use Among Suspected Malaria Episodes.

	March 2011 (Round 1)		December 2011 (Round 2)		March 2012 (Round 3)		Change Round 1 to Round 2		Change Round 1 to Round 3	
	Total n	% TakingACT	Total n	% TakingACT	Total n	% TakingACT	%	(p-value)	(p-value)	
	Among Households with a Suspected Malaria Episode									
Overall	187	54.55	217	57.6	307	67.75	3.06	−0.506	−0.0039	
*By Region*										
Rukwa	125	48	145	51.03	161	55.9	3.03	−0.623	−0.1675	
Mtwara	62	67.74	72	70.83	146	80.82	3.09	−0.6367	−0.0832	
	Among Individuals with a Suspected Malaria Episode									
Overall	229	51.09	308	54.55	468	61.97	3.45	−0.4346	−0.017	
Among Those Seeking Treatment	184	50.54	261	55.17	452	63.5	4.63	−0.3436	−0.0097	
Among Those Taking An Antimalarial	200	58.5	262	64.12	420	69.05	5.62	−0.2124	−0.0258	
*By Region*										
Rukwa	151	45.03	198	47.47	220	49.09	2.44	−0.6842	−0.5077	
Mtwara	78	62.82	110	67.27	248	73.39	4.45	−0.4736	−0.1111	
*By Sector in Which Treatment Sought*										
Public Sector	96	64.58	132	64.39	205	69.76	−0.19	−0.9771	−0.4502	
Private Sector	19	52.63	17	35.29	28	39.29	−17.3	−0.296	−0.4342	
Retail Sector	68	30.88	109	48.62	217	61.29	17.74	−0.0368	0	
*By Age*										
Under 5 Year Olds	60	48.33	68	42.65	91	57.14	−5.69	−0.53	−0.2709	
5 and Older	169	52.07	240	57.92	377	63.13	5.85	−0.2351	−0.0228	
*By SES*										
Bottom Tertile	69	47.83	74	54.05	159	61.01	6.23	−0.4733	−0.0977	
Middle Tertile	76	52.63	114	57.89	166	65.06	5.26	−0.5217	−0.1215	
Upper tertile	84	52.38	120	51.67	143	59.44	−0.71	−0.935	−0.4474	

### Ethical Approval

Ethical approval for the study was granted from the Harvard School of Public Health and from the Medical Research Coordinating Committee of the National Institute for Medical Research, Tanzania. Written consent to participate in the study was obtained from the primary female caregiver.

## Results

### Suspected Malaria Episodes

Suspected malaria episodes experienced in the four weeks prior to survey administration are presented in Table 1. In Round 1, roughly 6% of individuals (25% of households) report experiencing a suspected malaria episode. This percentage increased in each round and was consistently higher in Mtwara than in Rukwa and higher for children under five than for other ages. Pooling all rounds together, the fraction of children under five with a suspected malaria episode was 20% in Mtwara and 10.47% in Rukwa, similar to the most recent under five malaria burden estimates based on the Malaria Indicator Survey data collected from October 2007 to February 2008 in Mtwara (17.96%) and Rukwa (6.62%) [22].

### Treatment Seeking for Suspected Malaria Episodes

**Figure 3:**
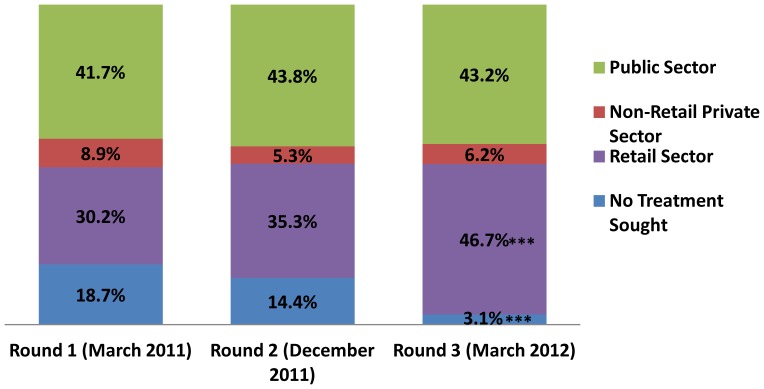
Percentage of Suspected Malaria Episodes Treated by Sector, March 2011–March 2012.

The public sector (public clinics and hospitals) was the most common place for treatment of suspected malaria (Figure 3), with 41.7% of episodes treated in Round 1. The retail sector (ADDOs and pharmacies) was the second most common treatment location (30.2% of episodes treated), followed by the episodes where no treatment was sought (18.7%) and, finally, treatment in the non-retail private sector (8.9%). Treatment seeking in the public sector and the non-retail private sector was basically unchanged, with no significant changes across survey rounds. Retail sector treatment seeking increased across each round, with most of the increase due to a decrease in individuals seeking no treatment at all. These changes were not significant between Rounds 1 and 2, but between Rounds 1 and 3 retail sector treatment seeking increased by 16.5 percentage points (p = .0009) and “no treatment” decreased by 15.6 percentage points (p = .0001).

### Trends in ACT Usage

ACT use for suspected malaria episodes is presented in Table 2. In March 2011, 54.6% of households experiencing a suspected malaria episode treated with ACT. Among those households with at least one suspected malaria episode, the proportion treating at least one of those episodes with ACT increased by 13.2 percentage points (roughly 24%) between Rounds 1 and 3 (p = .004). Increases in ACT taking of similar magnitudes are seen between Rounds 1 and 3 among all individual episodes (p = .017), among all episodes in which treatment was sought (p = .01) and in which any antimalarial was taken (p = .026). Point estimates for changes in ACT use between Rounds 1 and 2 were positive for these measures but none of these estimates were significant. ACT usage was consistently lower in Rukwa than Mtwara. Changes in ACT use in Rukwa were modest and insignificant, while the change in Mtwara between Rounds 1 and 3 was substantial at 10.55 percentage points among individuals and 13.08 among households, though not quite significant at conventional levels (p = .111 for individuals, p = .083 for households).

By far the greatest increases in ACT use were found among those individuals who sought treatment in the retail sector, where ACT usage increased from 30.9% in Round 1 to 48.6% in Round 2 (p = .037) and to 61.3% in Round 3 (p<.0001). Nearly 65% of suspected malaria episodes treated in the public sector in Round 1 were given ACTs, a rate that did not change significantly across any round. ACT taking for suspected malaria episodes increased by 11 percentage points between Rounds 1 and 3 for patients five and older (p = .023), though the change between Rounds 1 and 2 was not significant. In Round 1, rates of ACT taking–although lowest for the bottom tertile at 48%–did not differ greatly across SES tertiles. The biggest changes in ACT use were for the bottom and middle tertiles, at roughly 13 percentage points between Round 1 and Round 3, but this increase was not quite significant at conventional significance levels (p = .098 for bottom tertile, p = .122 for middle tertile).

### Preference for and Perceived Availability of ACTs

Respondents were asked several questions about antimalarial preference for themselves and for a child under five. They were first asked simply about their preferred antimalarial. They were then asked how often their preferred antimalarial was available if needed. Finally they were asked which antimalarial they preferred if money were no concern. In Round 1, just over half responded that ACT was preferred for themselves and a similar fraction preferred ACT if money were no concern (Table 3). Between Round 1 and Round 2, while the fraction of respondents who stated that they preferred ACT for themselves did not change significantly, the fraction who stated a preference for ACT if money were no concern increased by over 8 percentage points (p = .009). Between Round 1 and Round 3, both measures of preference for ACT increased significantly, with the simple preference increasing by 7.5 percentage points (p = .003) and the “if money were no concern” preference increasing by over 14 percentage points (p<.0001). When asked how often ACTs were available if needed, the fraction of people who replied “rarely/never” decreased by roughly 8 percentage points (p = .004) and 13 percentage points (p<.0001) between Rounds 1 and 2 and Round 1 and 3, respectively, with an associated increase in the fraction of respondents saying “always/most often” and “sometimes”. This pattern of results suggests that knowledge about the superior effectiveness and the increased availability of ACTs increased faster than knowledge about the reduction in price due to the subsidy. While increases in perceived availability of ACTs for a child were very similar (in magnitude and statistical significance) to those for the respondent, the significant increases in preference for ACT for a child were all found between Rounds 1 and 3 and none between Rounds 1 and 2.

**Table 3:** Antimalarial Preferences.

	March 2011 (Round 1)		December 2011 (Round 2)		March 2012 (Round 3)		Change Round 1 to Round 2		Change Round 1 to Round 3		
	Total n	Mean (%)	Total n	Mean (%)	Total n	Mean (%)	%	(p-value)	%		(p-value)
*Preferred Antimalarial (For Self)*											
ACT	741	53.85	692	55.2	725	61.38	1.36	−0.611	7.53	−0.0032	
Non-Artemisinin Antimalarial	741	42.24	692	42.2	725	35.03	−0.04	−0.9864	−7.21	−0.0066	
*Among those Preferring ACT: “How often is your preferred antimalarial available?”*											
Always/Most Often	398	73.12	380	69.74	444	77.7	−3.38	−0.3717	4.59	−0.2836	
Sometimes	398	9.3	380	20.53	444	18.47	11.23	−0.0002	9.17	−0.0021	
Rarely/Never	398	17.59	380	9.74	444	3.83	−7.85	−0.0042	−13.76	0	
*“If Money were no concern, which antimalarial would you buy for yourself?”*											
ACT	732	50.82	675	59.26	698	65.19	8.44	−0.0085	14.37	0	
Non-Artemisinin Antimalarial	732	45.77	675	39.26	698	33.81	−6.51	−0.0357	−11.95	−0.0001	
*Preferred Antimalarial (For a Child)*											
ACT	738	62.74	689	61.1	711	69.9	−1.63	−0.6077	7.16	−0.0053	
Non-Artemisinin Antimalarial	740	34.46	689	37.88	711	28.55	3.42	−0.273	−5.91	−0.0237	
*Among those Preferring ACT for a Child: “How often is your preferred antimalarial available?”*											
Always/Most Often	462	75.76	418	72.01	496	79.44	−3.75	−0.2663	3.68	−0.2548	
Sometimes	459	8.28	418	20.33	496	17.54	12.06	0	9.26	−0.0003	
Rarely/Never	462	16.02	418	7.42	496	3.02	−8.6	−0.0003	−12.99	0	
*“If Money were no concern, which antimalarial would you buy for a child?”*											
ACT	730	60.96	681	57.56	675	71.11	−3.4	−0.3104	10.15	−0.0003	
Non-Artemisinin Antimalarial	730	36.71	681	37.3	675	28.44	0.59	−0.8539	−8.27	−0.002	

Notes: Household level results take all households with at least one reported malaria episode and estimate the fraction of those households that treat at least one episode with ACT (either overall or by region).

## Discussion

This paper provides evidence on trends in ACT usage, treatment seeking for suspected malaria and patient preferences for ACTs over the first 15 months of the AMFm. These results offer critical, complementary evidence to the independent evaluation of the AMFm, which has been unable to examine ACT usage by individuals [18], [19]. In addition to capturing trends in ACT use during rapid increases in retail sector ACT availability, the data in this paper speak to the AMFm’s impact in remote areas. This is of particular interest because of the concern that a private sector approach to scaling up ACT coverage could have limited impact in communities with the lowest ability to pay and the least profit potential. Results presented here suggest that ACT use for suspected malaria increased significantly during the AMFm scale up in Tanzania. While the overall increase in ACT use over this 15 month period was moderate at roughly 13 percentage points (24 percent), this increase should be viewed in light of standard take-up rates of new products in private markets. In a review of both public health programs’ and private manufacturers’ experience with introduction of new products into developing country markets, Yamey et al. (2012) concluded that a 5–10 percentage point increase in usage after one year is considered a success [26]. The shifting to ACT purchases in the retail sector reported here was particularly substantial, nearly doubling from 31% to 61%. As retail sector patients were much less likely to take ACTs than public sector patients initially (31% vs. 65% at baseline), the AMFm appears to have substantially narrowed the gap in the likelihood of taking ACTs between treatment seekers in these sectors. These results also demonstrate an increase in the perception that ACTs are widely available and an increasing trend in preference for ACTs to treat both adults and children, though it is hard to say whether the latter is the cause or result of the increasing availability of low-priced ACTs.

A central concern about the AMFm is that it could divert patients from public sector care, where they would have been more likely to receive proper diagnosis and treatment. These results do not show evidence of public sector crowd out, but rather suggest a shift from “doing nothing” to retail sector antimalarial purchases. Another concern was that the AMFm might only benefit higher SES households. These results suggest on the contrary that the biggest increases in ACT use were for the bottom and middle SES tertiles though these changes were just below conventional significance levels. These results largely agree with those from several ACT subsidy pilots and from the paper by Fink et al. (2013) on the AMFm in Uganda. Fink et al (2013) find a 14 percentage point increase in ACT use among all patients taking antimalarials during the first year of the AMFm pilot in Uganda [27]. They also find that the largest increases in ACT use were among the poorest households and find a significant increase in ACT use among children under five with fever. Cohen et al. (2012) piloted an ACT subsidy in Western Kenya and found the biggest increases in ACT use among low SES households and found a shift from “doing nothing” to retail sector treatment for malaria [28]. Cohen et al. (2012) did find, however, some crowd out of public sector treatment among higher SES households. Kangwana et al. (2011) pilot an ACT subsidy for children in Kenya and also found no evidence of public sector crowd out or significant differences in ACT uptake across SES quintiles [29]. Sabot et al. (2009) pilot the ACT subsidy in Tanzania and also found that the increase in ACT use was not significantly different across low and high SES households [30].

A primary objective of the AMFm was to increase ACT use among children under five with fever. No significant increase in ACT use for suspected malaria among children was found here, although the sample size among this subgroup was too small to detect moderate increases in ACT use. A significant increase in ACT use for suspected malaria among people over age five was found. While less of a mortality concern, an increase in appropriate treatment among this age group is also important from an economic and public health perspective. For example, *Plasmodium* infection among school age children can affect performance and cognition [31], [32], malaria directly affects economic and labor productivity [33]-[36] and there is some recent, controversial evidence suggestive of higher malaria mortality among this age group than previously believed [37]. As younger children become increasingly protected by interventions such as insecticide-treated bed net distributions, older children are becoming more important disease reservoirs in some contexts [38], [39].

ACT use increased more in Mtwara than in Rukwa, possibly due to somewhat lower availability of ACTs in Rukwa (see Figure 2 and Yadav et al. 2012). Or another factor–such as Rukwa’s comparative remoteness and/or apparently lower prevalence–could drive both lower levels of ACT availability and use.

It is important to note that, while the changes in ACT use between Rounds 1 and 2 were almost consistently positive, they were often insignificant, whereas increases in ACT use by Round 3 were larger and more significant, despite the fact that ACT stocking was quite high by Round 2 (Figure 2). This is likely due in part to the more limited power to detect moderate changes between Rounds 1 and 2 (discussed in the methods section above) and also in part to a lag in the translation of increases in subsidized ACT stocking into increases in ACT use. This lag could be caused by the time it takes for patients or caregivers to become aware of the increased availability of ACTs at affordable prices (e.g. through word-of-mouth) and the dynamics of product adoption and diffusion, where an initial number of patients/caregivers who use subsidized ACTs slowly pass on knowledge about their efficacy, availability and price to the remaining population [40], [41].

These results should be interpreted with a number of caveats. First, the data are drawn from only two regions in Tanzania, purposefully selected for their remoteness, and will have to be viewed in light of other research on the AMFm to gauge their generalizability. Second, the presence of ADDOs could have made these regions of Tanzania particularly favorable for ACT distribution in the retail sector. Third, the power to detect significant changes in ACT use among subgroups and the more modest changes estimated between Rounds 1 and 2 was limited by sample size, as the reported rates of suspected malaria were somewhat lower than expected based on the 2007–2008 MIS. An analysis that purposively sampled higher malaria risk households could have explored trends in ACT use among sub-groups with more accuracy.

A number of considerations arise from the measurement of suspected, but in many cases unconfirmed, malaria in the survey tool. Without local parasite prevalence data spanning the period of study, it is difficult to gauge the extent to which the increase in suspected malaria episodes reported across rounds reflects a true increase in malarial infections or rather an increase in the belief that malaria-like illnesses are actually malaria. It is possible that perceptions about the likelihood that an illness was malaria increased as a result of the AMFm. If many of these cases are not truly malaria, increases in ACT use are not necessarily desirable. On the other hand, if many true malaria cases are “missed” because, for example, caregivers do not recognize symptoms, than an increase in the recognition of malarial illness should have a positive effect on morbidity. Since suspected malaria (regardless of symptoms) is what triggers treatment seeking, it was important for this study to utilize a broad, self-reported malaria measure, but this may limit the extent to which these results can be compared to estimates from MIS surveys, as the latter ask about treatment seeking for fever episodes among children under five. Finally, it is possible that caregivers may misremember episodes or how they were treated, but this measurement error should not have changed systematically across survey rounds, so an analysis of trends in ACT usage should not be compromised.

An important concern in interpreting these results stems from the absence of a control group or counterfactual policy environment. Even in the absence of the AMFm, ACT use could have been increasing over time in Tanzania, for example if awareness of ACT effectiveness or private sector competition increased. This would lead to an overstatement of the role of the AMFm. It is questionable how much the increase in ACT use found here was driven by non-AMFm factors, however, as those factors would not have removed a major barrier to ACT adoption–namely, the high price of unsubsidized ACTs. While it is possible that the opportunity to receive a blood-based diagnosis was increasing for public sector patients during this period, this is unlikely to have driven the results we find here since no significant increase in public sector treatment seeking or ACT taking among public sector patients was found.

These results could also be understating the impact of the AMFm, as data collection began several months after the first–albeit small (.25 million doses)–batch of subsidized ACTs arrived in Tanzania and ended before the conclusion of the AMFm. Finally, these results cannot be seen as the result of a completely unrestricted market for subsidized ACTs as, starting September 2011, the AMFm Secretariat began using a “rationing mechanism”, approving only a fraction of private sector orders for co-payments on subsidized ACTs [18].

A factory-gate subsidy for ACTs appears to have been effective at increasing usage of recommended malaria treatment, even in poor, remote communities. Firm conclusions about the impact of the AMFm in Tanzania or more broadly cannot be drawn from these findings given their geographic restrictions. However, these results are cause for optimism that the substantial, national-scale increases in ACT availability and affordability attributed to the AMFm that have been reported elsewhere are contributing to corresponding increases in usage of the drugs and therefore progress towards the achievement of national and global goals of widespread, prompt use of ACT treatment.

## Supporting Information

Table S1 **Characteristics of Sampled Households across Regions and Rounds.**
(DOCX)

## References

[1] World Health Organization (2010) Guidelines for the Treatment of Malaria. Geneva, Switzerland: World Health Organization.

[2] Bhattarai A, Ali AS, Kachur SP, Märtensson A, Abbas AK, et al (2007) Impact of artemisinin-based combination therapy and insecticide-treated nets on malaria burden in Zanzibar. PLoS Medicine 4(11): e309. 17988171 10.1371/journal.pmed.0040309 PMC2062481

[3] Otten M, Aregawi M, Were W, Karema C, Medin A, et al (2009) Initial evidence of reduction of malaria cases and deaths in Rwanda and Ethiopia due to rapid scale-up of malaria prevention and treatment. Malaria Journal 8: 14. 19144183 10.1186/1475-2875-8-14 PMC2653503

[4] O’Connell KA, Gatakaa H, Poyer S, Njogu J, Evance I, et al (2011) Got ACTs?: Availability, price, market share and provider knowledge of anti-malarial medicines in public and private sector outlets in six malaria-endemic countries. Malaria Journal 10: 326. 22039838 10.1186/1475-2875-10-326 PMC3227612

[5] Patoillard E, Hanson KG, Goodman CA (2010) Retail sector distribution chains for malaria treatment in the developing world: a review of the literature. Malaria Journal 9: 50. 20149246 10.1186/1475-2875-9-50 PMC2836367

[6] Talisuna A, Grewal P, Rwakimari JB, Mukasa S, Jagoe G, et al (2009) Cost is killing patients: subsidizing effective antimalarials. The Lancet 374(9697): 1224–1226. 10.1016/S0140-6736(09)61767-0 19819377

[7] Kangwana BB, Njogu J, Wasunna B, Kedenge SV, Memusi DN, et al (2009) Malaria drug shortages in Kenya: a major failure to provide access to effective treatment. American Journal of Tropical Medicine and Hygiene 80(5): 737–738. 19407116 PMC2679204

[8] Zurovac D, Ndhlovu M, Sipilanyambe N, Sipilanyambe N, Chanda P, et al (2007) Pediatric malaria case-management with artemether-lumefantrine in Zambia: a repeat cross-sectional study. Malaria Journal 6: 31. 17367518 10.1186/1475-2875-6-31 PMC1832199

[9] Zurovac D, Tibenderana JK, Nankabirwa J, Ssekitooleko J, Njogu JN, et al (2008) Malaria case-management under artemether-lumefantrine treatment policy in Uganda. Malaria Journal 7: 181. 18803833 10.1186/1475-2875-7-181 PMC2556699

[10] Sudoi RK, Githinji S, Nyandigisi A, Muturi A, Snow RW, et al (2012) The magnitude and trend of artemether-lumefantrine stock-outs at public health facilities in Kenya. Malaria Journal 11: 37. 22316236 10.1186/1475-2875-11-37 PMC3306750

[11] Onwujekwe O, Hanson K, Uzochukwu B, Ezeoke O, Eze S, et al (2010) Geographic inequities in provision and utilization of malaria treatment services in southeast Nigeria: Diagnosis, providers and drugs. Health Policy 94(2): 144–149. 19836852 10.1016/j.healthpol.2009.09.010

[12] Cohen JM, Sabot O, Sabot K, Gordon M, Gross I, et al (2010) A pharmacy too far? Equity and spatial distribution of outcomes in the delivery of subsidized artemisinin-based combination therapies through private drug shops. BMC Health Services Research 10 (Suppl 1)S6. 20594372 10.1186/1472-6963-10-S1-S6 PMC2895750

[13] The Global Fund to Fight AIDS, Tuberculosis and Malaria (2010) Global Fund quality assurance policy for pharmaceutical products. Twenty-second Board Meeting, Sofia, 13–15 Dec 2010. GF-B22–11, Revision 1, Annex 1.

[14] Adeyi O, Atun R (2010) Universal access to malaria medicines: innovation in financing and delivery. The Lancet 376(9755): 1869–1871. 10.1016/S0140-6736(10)61189-0 20940074

[15] Laxminarayan R, Gelband H (2009) A Global Subsidy: Key to Affordable Drugs for Malaria? Health Affairs 28(4): 949–961. 19597193 10.1377/hlthaff.28.4.949

[16] World Health Organization (2011) World Malaria Report. Geneva, Switzerland: World Health Organization.

[17] Arrow K, Panosian C, Gelband H, editors. (2004) Saving Lives, Buying Time: Economics of Malaria Drugs in an Age of Resistance. Washington D.C.: Institute of Medicine, National Academies Press. 25009879

[18] AMFm Independent Evaluation Team (ICF International and London School of Hygiene and Tropical Medicine) (2012) Independent Evaluation of Phase 1 of the Affordable Medicines Facility-malaria (AMFm). Available: http://www.theglobalfund.org/en/amfm/independentevaluation/. Accessed 2013 Jul 2.

[19] Tougher S, Yazoume Y, Amuasi JH, Kourgueni IA, Thomson R, et al (2012) Effect of the Affordable Medicines Facility-malaria (AMFm) on the availability, price, and market share of quality-assured artemisinin-based combination therapies in seven countries: a before-and-after analysis of outlet survey data. The Lancet 380(9857): 1916–1926. 10.1016/S0140-6736(12)61732-2 23122217

[20] Kamal-Yanni M (2010) Affordable Medicines Facility for malaria: reasonable or rash? The Lancet 375(9709): 121. 10.1016/S0140-6736(10)60048-7 20109888

[21] Bitran R, Martorell B. (2009) AMFm: Reaching the poorest of the poor with effective malaria drugs. *Resources for the Future Discussion Paper* Available: http://www.rff.org/Publications/Pages/PublicationDetails.aspx?PublicationID=20793. Accessed 2013 Jul 2.

[22] Gosoniu L, Msengwa A, Lengeler C, Vounatsou P (2012) Spatially explicit burden estimates of malaria in Tanzania: bayesian geostatistical modeling of the malaria indicator survey data. *PLoS ONE* 2012 7(5): e23966. 10.1371/journal.pone.0023966 PMC3359352 22649486

[23] Ministry of Planning, Economy and Empowerment (2006) *National Population Policy*. Dar es Salaam, Tanzania.

[24] Yadav P, Cohen J, Alphs S, Arkedis J, Larson PS, et al (2012) Trends in availability and prices of subsidized act over the first year of the AMFm: evidence from remote regions in Tanzania. Malaria Journal 11: 299. 22929587 10.1186/1475-2875-11-299 PMC3502171

[25] Filmer D, Pritchett LH (2001) Estimating wealth effects without expenditure data–or tears: an application to educational enrollments in states of India. Demography 38: 115–132. 11227840 10.1353/dem.2001.0003

[26] Yamey G, Schaferhoff M, Montagu D (2012) Piloting the Affordable Medicines Facility-malaria: what will success look like? Bulletin of the WHO 90: 452–460. 10.2471/BLT.11.091199 PMC3370360 22690035

[27] Fink G, Dickens WT, Jordan M and Cohen JL (2013) Access to Subsidized ACT and malaria treatment–evidence from the first year of the AMFm program in six districts in Uganda. Health Policy and Planning; doi: 10.1093/heapol/czt041. 10.1093/heapol/czt041 23783833

[28] Cohen J, Dupas P, Schaner S (2012) Price subsidies, diagnostic tests and targeting of malaria treatment: evidence from a randomized controlled trial. *National Bureau of Economic Research,* Working Paper #17943. Available: http://www.nber.org/papers/w17943. Accessed 2013 Jul 2.

[29] Kangwana BP, Kedenge SV, Noor AM, Alegana VA, Nyandigisi AJ, et al (2011) The Impact of Retail-Sector Delivery of Artemether–Lumefantrine on Malaria Treatment of Children under Five in Kenya: A Cluster Randomized Controlled Trial. PLoS Medicine 8(5): e1000437. 21655317 10.1371/journal.pmed.1000437 PMC3104978

[30] Sabot OJ, Mwita A, Cohen JM Ipuge Y, Gordon M, et al (2009) Piloting the global subsidy: the impact of subsidized artemisinin-based combination therapies distributed through private drug shops in rural Tanzania. *PLoS One* 4: e6857. 19724644 10.1371/journal.pone.0006857 PMC2730578

[31] Halliday KE, Karanja P, Turner EL (2012) Plasmodium falciparum, anaemia and cognitive and educational performance among school children in an area of moderate malaria transmission: baseline results of a cluster randomized trial on the coast of Kenya. Tropical Medicine and International Health 17(5): p.532. 10.1111/j.1365-3156.2012.02971.x PMC3506732 22950512

[32] Fink G, Olgiati A, Hawela M, Miller JM, Matafwali B (2013) Association between early childhood exposure to malaria and children’s pre-school development: evidence from the Zambia early childhood development project. *Malaria Journal* 12: 12. 23297692 10.1186/1475-2875-12-12 PMC3546841

[33] Chima R.I., Goodman CA, Mills A (2003) The economic impact of malaria in Africa: a critical review of the evidence. Health Policy. 63(1): p.17–36. 10.1016/s0168-8510(02)00036-2 12468115

[34] Asenso-Okyere K., Asanta FA, Tarekegn J, Andam KS (2011) A review of the economic impact of malaria in agricultural development. *Agricultural Economics*. 42(3): p.293–304.

[35] Goodman CA, Coleman P, Mills A (2000) Economic analysis of malaria control in sub-Saharan Africa. *Global forum for health research: promoting research to improve the health of the poor*. Geneva, Switzerland.

[36] Gallup JL, Sachs JD (2001) The Economic Burden of Malaria. American Journal of Tropical Medicine and Hygiene. 64(1–2 Suppl): 85–96. 10.4269/ajtmh.2001.64.85 11425181

[37] Murray CJ, Rosenfeld LC, Lim SS, Andrews KG, Foreman KJ, et al (2012) Global malaria mortality between 1980 and 2010: a systematic analysis. The Lancet 379: 413–431. 10.1016/S0140-6736(12)60034-8 22305225

[38] Noor AM, Kirui VC, Brooker SJ, Snow RW (2009) The use of insecticide treated nets by age: implications for universal coverage in Africa. *BMC Public Health*. 9: p.369. 10.1186/1471-2458-9-369 PMC2761895 19796380

[39] Mawili-Mboumba DP, Akotet MKB, Kendjo E, Nzamba J, Medang MO, et al (2013) Increase in malaria prevalence and age of at risk population in different areas of Gabon. Malaria Journal 12: 3. 23282198 10.1186/1475-2875-12-3 PMC3549767

[40] Cook AG (2006) Forecasting for the Pharmaceutical Industry: Models for New Product and In-Market Forecasting and How to Use Them. Aldershot, England: Gower Publishing.

[41] Rogers EM (1962) Diffusion of Innovations. New York: The Free Press.

