# Supplementary material for: Do Price Subsidies on Artemisinin Combination Therapy for Malaria Increase Household Use?: Evidence from a Repeated Cross-Sectional Study in Remote Regions of Tanzania
Source: PLoS One. 2013 Jul 29;8(7):e70713. doi: 10.1371/journal.pone.0070713 (PMC3726608; doi:10.1371/journal.pone.0070713)
Supplement: Table S1 — Characteristics of Sampled Households across Regions and Rounds. (DOCX) [file pone.0070713.s001.docx]

| Table S1. Characteristics of Sampled Households across Regions and Rounds | | | | | | | | |  |  |
| --- | --- | --- | --- | --- | --- | --- | --- | --- | --- | --- |
|  | **March 2011 (Round 1)** | |  | **December 2011 (Round 2)** | |  | **March 2012 (Round 3)** | |  |  |
|  | Mtwara | Rukwa |  | Mtwara | Rukwa |  | Mtwara | Rukwa |  |  |
| n | 264 | 500 |  | 264 | 492 |  | 264 | 492 |  |  |
| *Religion* |  |  |  |  |  |  |  |  |  |  |
| Catholic | 0.76% | 73.80% |  | 2.65% | 69.72% |  | 1.52% | 71.75% |  |  |
| Protestant | 1.52% | 3.60% |  | 3.41% | 3.86% |  | 2.27% | 2.44% |  |  |
| Born Again |  | 2.60% |  |  | 3.66% |  |  | 3.86% |  |  |
| Other Christian | 2.27% | 9.40% |  | 0.76% | 13.21% |  | 0.76% | 13.41% |  |  |
| Muslim | 95.45% | 5.40% |  | 93.18% | 5.08% |  | 95.08% | 3.25% |  |  |
| Seventh Day Adventist |  | 2.20% |  |  | 2.85% |  | 0.38% |  |  |  |
| Traditional |  | 1% |  |  | 1.63% |  |  | 3.05% |  |  |
| Non-religious |  | 1.80% |  |  |  |  |  | 0.61% |  |  |
| Other |  | 0.20% |  |  |  |  |  | 1.42% |  |  |
| *Language* |  |  |  |  |  |  |  |  |  |  |
| Fipa |  | 67.20% |  |  | 65.65% |  |  | 64.63% |  |  |
| Makonde | 82.58% |  |  | 81.82% |  |  | 82.58% |  |  |  |
| *Marital Status* |  |  |  |  |  |  |  |  |  |  |
| Married, monogamous | 55.30% | 71.40% |  | 48.11% | 66.87% |  | 55.30% | 65.71% |  |  |
| Married, polygamous | 20.83% | 14.60% |  | 21.21% | 17.07% |  | 18.94% | 16.53% |  |  |
| Widowed | 3.41% | 7.60% |  | 15.91% | 6.91% |  | 8.71% | 7.35% |  |  |
| Divorced/separated | 13.64% | 4.40% |  | 7.95% | 3.05% |  | 10.98% | 5.51% |  |  |
| Cohabitating |  |  |  | 1.14% | 0.41% |  | 1.14% |  |  |  |
| Single | 6.82% | 2% |  | 5.68% | 5.69% |  | 4.92% | 4.90% |  |  |
| *Literacy* |  |  |  |  |  |  |  |  |  |  |
| Read Swahili | 68.56% | 71.60% |  | 59.09% | 65.85% |  | 70.08% | 62.60% |  |  |
| Write Swahili | 67.42% | 71% |  | 58.71% | 65.24% |  | 68.56% | 61.79% |  |  |
| *Number of Children* |  |  |  |  |  |  |  |  |  |  |
| Mean | 4.56 | 5.71 |  | 4.3 | 5.73 |  | 4.22 | 5.75 |  |  |
| Median | 4 | 5 |  | 4 | 5 |  | 4 | 5 |  |  |
| *Household Size* |  |  |  |  |  |  |  |  |  |  |
| Mean | 4.27 | 5.64 |  | 4.08 | 5.64 |  | 4.54 | 5.56 |  |  |
| Median | 5.64 | 5 |  | 4 | 5 |  | 4 | 5 |  |  |
| *Water Source* |  |  |  |  |  |  |  |  |  |  |
| Piped into dwelling |  |  |  |  | 0.20% |  | 0.38% |  |  |  |
| Piped into plot/yard | 0.76% | 0.80% |  | 0.76% | 1.02% |  | 0.38% | 2.03% |  |  |
| Public tap | 15.53% | 23.80% |  | 12.50% | 32.93% |  | 17.80% | 10.37% |  |  |
| Borehole/well | 4.17% | 8.20% |  | 15.53% | 2.24% |  | 18.94% | 1.42% |  |  |
| Protected well | 14.39% | 7.80% |  | 14.39% | 5.08% |  | 1.14% | 30.08% |  |  |
| Unprotected well | 9.47% | 17% |  | 6.44% | 20.33% |  | 4.92% | 12.60% |  |  |
| Rain water | 32.95% | 0.60% |  | 34.09% | 0.61% |  | 44.70% | 0.20% |  |  |
| Surface water | 21.59% | 41.80% |  | 14.77% | 37.60% |  | 10.98% | 42.89% |  |  |
| Brought in | 1.14% |  |  | 1.52% |  |  | 0.76% |  |  |  |
| *Toilet type* |  |  |  |  |  |  |  |  |  |  |
| Own flush toilet | 4.17% | 1.80% |  |  | 5.69% |  | 0.38% | 3.05% |  |  |
| Shared flush toilet | 0.38% | 2.80% |  |  | 1.83% |  | 0.38% | 2.85% |  |  |
| Own pit latrine | 91.29% | 80.20% |  | 95.45% | 71.95% |  | 93.94% | 77.85% |  |  |
| Shared pit latrine | 4.17% | 11.20% |  | 3.79% | 18.50% |  | 4.17% | 13.01% |  |  |
| Own ventilated improved pit latrine |  | 2% |  | 0.76% | 1.42% |  | 1.14% | 1.63% |  |  |
| Shared ventilated improved pit latrine |  | 0.80% |  |  | 0.20% |  |  | 0.20% |  |  |
| Bush/field/forest |  | 1.20% |  |  | 0.20% |  |  | 1.42% |  |  |
| *Cooking fuel* |  |  |  |  |  |  |  |  |  |  |
| Kerosene/paraffin | 0.38% |  |  |  |  |  |  |  |  |  |
| Charcoal | 9.85% | 27.40% |  | 8.33% | 24.59% |  | 5.68% | 25.81% |  |  |
| Firewood collected | 82.20% | 71.80% |  | 90.15% | 72.56% |  | 90.15% | 71.14% |  |  |
| Firewood purchased | 7.58% | 0.80% |  | 1.52% | 2.24% |  | 4.17% | 2.85% |  |  |
| *SES index components* |  |  |  |  |  |  |  |  |  |  |
| Asset, mobile phone (1+) | 56.44% | 39.40% |  | 64.77% | 46.75% |  | 65.15% | 45.93% |  |  |
| Asset, radio (1+) | 65.15% | 63.40% |  | 56.06% | 59.76% |  | 67.80% | 56.10% |  |  |
| Asset, bicycle (1+) | 68.18% | 47.80% |  | 57.20% | 45.93% |  | 68.56% | 46.75% |  |  |
| Ever watch television | 39.02% | 45.89% |  | 24.62% | 22.97% |  | 42.80% | 19.92% |  |  |
| Ever read newspaper | 23.11% | 33.87% |  | 22.73% | 16.87% |  | 25.76% | 14.02% |  |  |
| Ever listen radio | 89.02% | 80.36% |  | 79.17% | 76.63% |  | 79.17% | 76.63% |  |  |
| Daily radio listening | 60% | 53.37% |  | 35.89% | 42.97% |  | 52.68% | 36.17% |  |  |
| Read newspaper < once/wk | 52.46% | 66.86% |  | 23.33% | 63.86% |  | 44.12% | 62.32% |  |  |
| Cooking fuel, charcoal | 9.85% | 27.40% |  | 8.33% | 24.59% |  | 5.68% | 25.81% |  |  |
| Cooking fuel, collected wood | 82.20% | 71.80% |  | 90.15% | 72.56% |  | 90.15% | 71.14% |  |  |
| Own pit latrine | 91.29% | 80.20% |  | 95.45% | 71.95% |  | 93.94% | 77.85% |  |  |
| Treat own water | 16.67% | 34.40% |  | 15.15% | 31.30% |  | 15.15% | 27.85% |  |  |
| If own land, have title deed | 13.79% | 8.41% |  | 9.50% | 8.35% |  | 8.76% | 8.69% |  |  |
| Walls, baked brick | 7.95% | 65.40% |  | 18.56% | 80.89% |  | 10.98% | 76.42% |  |  |
| Walls, sundried brick/mud | 62.50% | 31.80% |  | 28.79% | 18.09% |  | 32.95% | 17.28% |  |  |
| Roof, iron sheets/tin | 64.39% | 54.40% |  | 59.85% | 52.85% |  | 64.39% | 54.47% |  |  |
| Roof, grass roof | 35.61% | 45.60% |  | 40.15% | 47.15% |  | 35.61% | 45.53% |  |  |
| Floor, finished (cement) | 24.24% | 25.20% |  | 22.35% | 26.83% |  | 22.35% | 27.44% |  |  |
| Floor, rudimentary wood) | 1.14% | 3.40% |  | 0.76% | 0.41% |  | 3.79% |  |  |  |
| Floor, natural (earth) | 74.62% | 71.40% |  | 76.89% | 72.56% |  | 73.86% | 72.36% |  |  |
| *Distance to nearest shop* |  |  |  |  |  |  |  |  |  |  |
| Travel Distace (km) | 0.55 | 1.05 |  | 0.34 | 0.78 |  | 0.27 | 0.83 |  |  |
| Walking Time (minutes) | 16.49 | 25.33 |  | 13.04 | 11.04 |  | 11.61 | 13.44 |  |  |
| *Number of bednets used last night* |  |  |  |  |  |  |  |  |  |  |
| Mean | 0.69 | 0.48 |  | 0.59 | 0.41 |  | 0.56 | 0.44 |  |  |
| 0 | 1.52% | 3% |  | 8.33% | 14.23% |  | 6.82% | 7.32% |  |  |
| 1 | 17.42% | 19% |  | 20.83% | 17.48% |  | 20.08% | 17.28% |  |  |
| 2 to 4 | 68.55% | 70.60% |  | 64.77% | 65.04% |  | 65.54% | 72.16% |  |  |
| 5 or more | 12.51% | 7.40% |  | 6.07% | 3.25% |  | 7.58% | 3.26% |  |  |
|  |  |  |  |  |  |  |  |  |  |  |
